# A Realistic Neural Mass Model of the Cortex with Laminar-Specific Connections and Synaptic Plasticity – Evaluation with Auditory Habituation

**DOI:** 10.1371/journal.pone.0077876

**Published:** 2013-10-30

**Authors:** Peng Wang, Thomas R. Knösche

**Affiliations:** Max Planck Institute for Human Cognitive and Brain Sciences, MEG and Cortical Networks, Leipzig, Germany; SUNY Downstate MC, United States of America

## Abstract

In this work we propose a biologically realistic local cortical circuit model (LCCM), based on neural masses, that incorporates important aspects of the functional organization of the brain that have not been covered by previous models: (1) activity dependent plasticity of excitatory synaptic couplings via depleting and recycling of neurotransmitters and (2) realistic inter-laminar dynamics via laminar-specific distribution of and connections between neural populations. The potential of the LCCM was demonstrated by accounting for the process of auditory habituation. The model parameters were specified using Bayesian inference. It was found that: (1) besides the major serial excitatory information pathway (layer 4 to layer 2/3 to layer 5/6), there exists a parallel “short-cut” pathway (layer 4 to layer 5/6), (2) the excitatory signal flow from the pyramidal cells to the inhibitory interneurons seems to be more intra-laminar while, in contrast, the inhibitory signal flow from inhibitory interneurons to the pyramidal cells seems to be both intra- and inter-laminar, and (3) the habituation rates of the connections are unsymmetrical: forward connections (from layer 4 to layer 2/3) are more strongly habituated than backward connections (from Layer 5/6 to layer 4). Our evaluation demonstrates that the novel features of the LCCM are of crucial importance for mechanistic explanations of brain function. The incorporation of these features into a mass model makes them applicable to modeling based on macroscopic data (like EEG or MEG), which are usually available in human experiments. Our LCCM is therefore a valuable building block for future realistic models of human cognitive function.

## Introduction

Traditionally, two main classes of models have been commonly used to explore the dynamics of neural circuits [1]. One is based on single neuron simulation using spiking neuron models, for example, of the leaky integrate-and-fire or the more elaborate Hodgkin-Huxley types [2]–[3]. Such networks include multiple interconnected neurons and the short-term synaptic plasticity depends on the dynamics of the presynaptic spike trains [4]–[9]. There is an extensive literature on the use of such models to link detailed structural and physiological features, such as inter- and intralaminar connections, various neurotransmitter-receptor systems and synaptic plasticity mechanisms, to various brain functions, including perceptual binding, attention, learning and speech perception [10]–[17]. These models are, for example, relevant for single cell recordings in animals, while their state variables are not captured adequately by macroscopic measurements, like EEG, MEG, local field potentials (LFP), or functional magnetic resonance imaging (fMRI). In contrast, neural mass models (NMMs) [18]–[25] describe the mean activity of entire neural populations, represented by their averaged firing rates and membrane potentials. Such models are, therefore, more useful for modeling macroscopic brain signals. Despite their parsimony, NMMs are still biologically realistic; that is, their parameters are related to microscopically measurable quantities, such as dendritic time constants.

**Figure 1:**
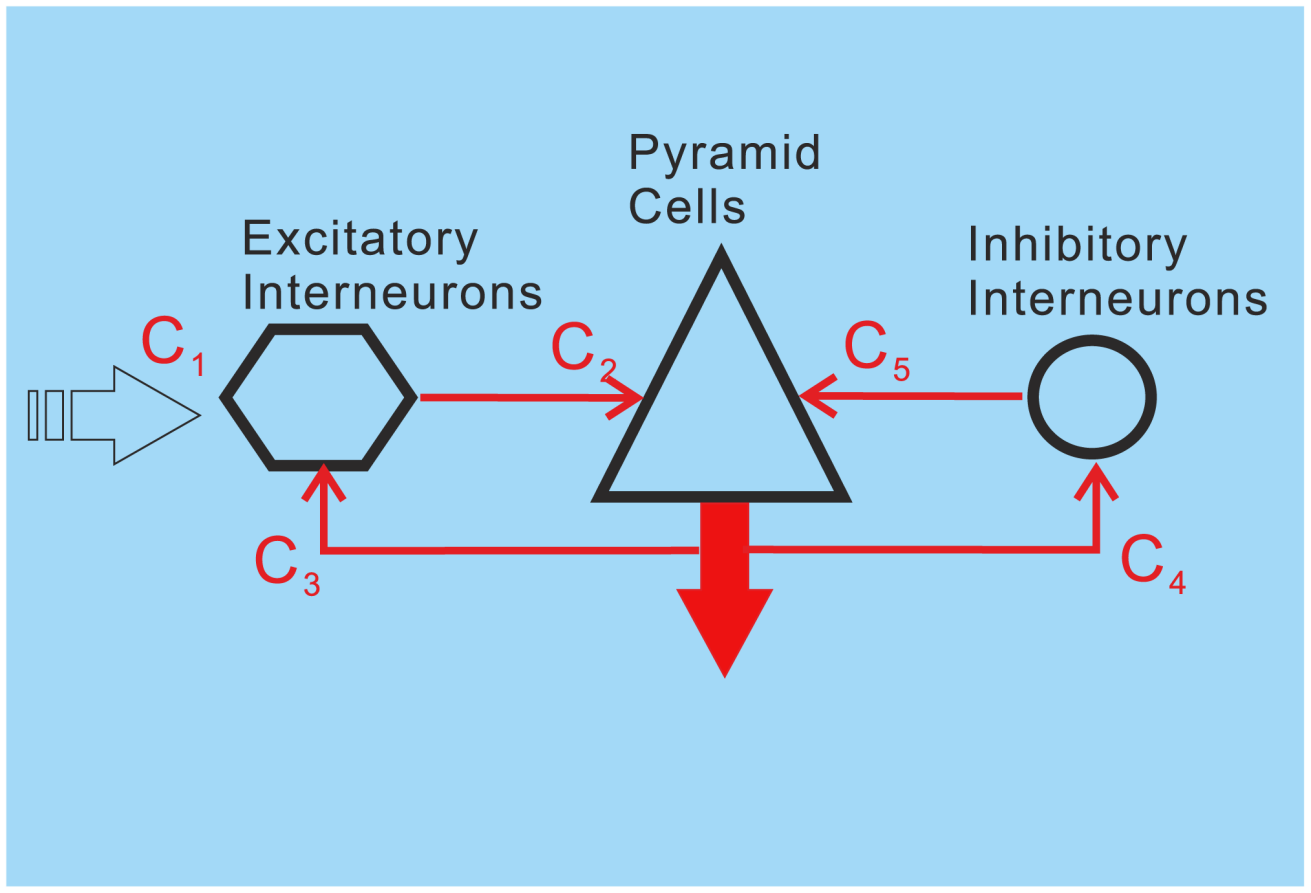
Neural mass model of Jansen and Rit with excitatory interneurons (EIN), pyramidal cells (PC) as well as inhibitory interneurons (IIN).

In the past, brain networks and functions have been investigated using NMMs with different sets of assumptions, e.g., by Wilson and Cowan [26], Freeman [20], Wright and Liley [27], Robinson and colleagues [28], Rennie and colleagues [29], Jansen and Rit, and Lopes da Silva and colleagues [21]–[22]. One of the most widely used ways to account for the dynamics of a cortical circuit has been the approach of Jansen and Rit [18]–[19], which comprises three interconnected neural populations: pyramidal cells (PCs), excitatory interneurons (EINs), and inhibitory interneurons (IINs) (Fig. 1). The averaged membrane potentials of the PCs are considered proportional to the observed EEG/MEG signals [30]. David and colleagues [31] added an inter-area connectivity scheme following the hierarchical rules described by Felleman and Van Essen [32], in order to assemble a network of coupled sources, Wendling and colleagues [33] separated the originally singular IIN population into a fast GABAergic and a slow GABAergic IIN, and Zavaglia and colleagues [34] added a recurrent loop to the circuit of fast GABAergic IINs. These models have been used to simulate various EEG/MEG features in both time and frequency domains, such as: brain rhythms ranging from the delta to the gamma bands [18], [34]–[35]; event-related evoked responses [31], [36]–[39], induced responses [25], [40]; spectral responses [41]–[43]; and epilepsy-like activity [33], [44]. Moreover these model have also been used to account for effects in other brain image modalities such as fMRI [45] and voltage sensitive dyes [46].

However, most of these approaches are based on static network structures with fixed connection strength, synaptic weights and time constants. This severely limits their potential to explain brain function, which is crucially dependent on plasticity. Accordingly, models of cortical networks consisting of several connected NMMs have been used to study the effect of short-term synaptic plasticity. In their epilepsy study, David and colleagues [47] simulated short-term plasticity as a fast modulation of synaptic efficacies in either intrinsic or extrinsic connections to the epileptic focus. Garrido and colleagues [39] modeled auditory repetition effects on connections within and between the sources in primary auditory cortex and superior temporal gyrus. However, in their model, they did not include any mechanistic model to constrain the plasticity process. Instead they simply mapped changing signal amplitudes to changes in connectivity.

Here, we intend to develop a mechanistic model for synaptic plasticity and use it to explain a specific phenomenon based on short-term synaptic plasticity – habituation. Habituation refers to the suppression of neural and behavioral responses as a result of repeated stimulation. It usually follows an exponential decay function of stimulus repetition and is reversed if stimulation changes [48]. This neural mechanism helps us to use our limited brain resources to interact with our environment in an efficient way: repeated irrelevant information will be ignored. Short-term habituation of event-related responses in electroencephalography (EEG) or magnetoencephalography (MEG) has been reported in the auditory domain [49]–[50]. The N100 and N100m are the most reliable and prominent peaks observed in the auditory evoked potential (AEP) and auditory evoked field (AEF), respectively, and appear about 100 ms after stimulus onset [51]. Repeated stimulation causes attenuation of the N100/N100m amplitude if the stimuli (e.g., short tones) are presented in rapid succession (e.g., with 500 ms spacing). This amplitude suppression recovers after about 6 to 10 s of stimuli free time [52]. This effect is of great interest in clinical neuroscience, because impaired habituation has been observed in patients suffering from, for example, schizophrenia [53], Alzheimer’s disease [54], or migraines [55]. In cognitive neuroscience, the neuronal adaptation in the auditory cortex is associated with the mismatch negativity. This is a negative EEG deflection in response to deviant stimuli and has been explained in terms of short-term habituation [56]–[57]. Its MEG counterpart is called the mismatch field [58].

The underlying neural mechanisms of short-term habituation, however, are still not fully understood. On a microscopic level, considerable insight has been gained from animal studies. In the 1970s Castellucci and Kandel [59]–[60] showed that synaptic modification might be a possible basis for habituation, based on their series of experiments of the aplysia gill-withdrawal reflex. They found that after habituation there were fewer synaptic vesicles released per action potential. Studies of frog neuromuscular junctions as well as hippocampal synapses in rats have, furthermore, suggested that a decrease in transmitter release can be caused by a depletion of the readily releasable pool of vesicles, or a decrease in the release probability of each docked vesicle, or both [61]–[63].

On a very different level of detail, there are a number of EEG and MEG studies that have shed light on the mechanisms of habituation. Garrido and colleagues [39] used human EEG and computational modeling techniques to suggest that the reduction of evoked responses is associated with a decrease of the connectivity within or between the involved cortical areas. In their MEG study, Rosburg and colleagues found that only the first repetition of auditory stimuli resulted in a decrease of the amplitude of the AEF [64]. There was no evidence for any further response decrement after the 2nd stimulus. The results suggested that the suppression of the AEF was probably due to the refractoriness of cell populations involved in the generation of AEF components. Todorovic and colleagues [65] demonstrated in their MEG auditory experiment that the reduction of the AEF was larger for expected repetitions than for unexpected ones and, thereby, provided evidence for a top-down prior expectation modulation of the habituation.

The question is: How can these different scales of description be linked together? That is, how do we construct a comprehensive model of neural circuits that can capture important aspects of the microscopic generative mechanism of short-term habituation and, at the same time, predict macroscopic effects like N100 or N100m amplitude reduction? Such a comprehensive model would allow for data from different sources, both macroscopic and microscopic, to be integrated and enable testing of hypotheses and quantification of microscopic dynamics for given macroscopic observations [42].

In our approach, we model the habituation as a function of the dynamic change in average firing rate. We associate the synaptic connection strength with the neuronal vesicles’ release probability. Repetition of stimuli causes insufficient availability of vesicles in releasing pools and reduces the release probability, which in turn causes a reduction in synaptic connection strength and hence EEG/MEG signal amplitude. The recovery from habituation is linked to the process of recycling these vesicles back to the releasing pools, which occurs spontaneously.

**Figure 2:**
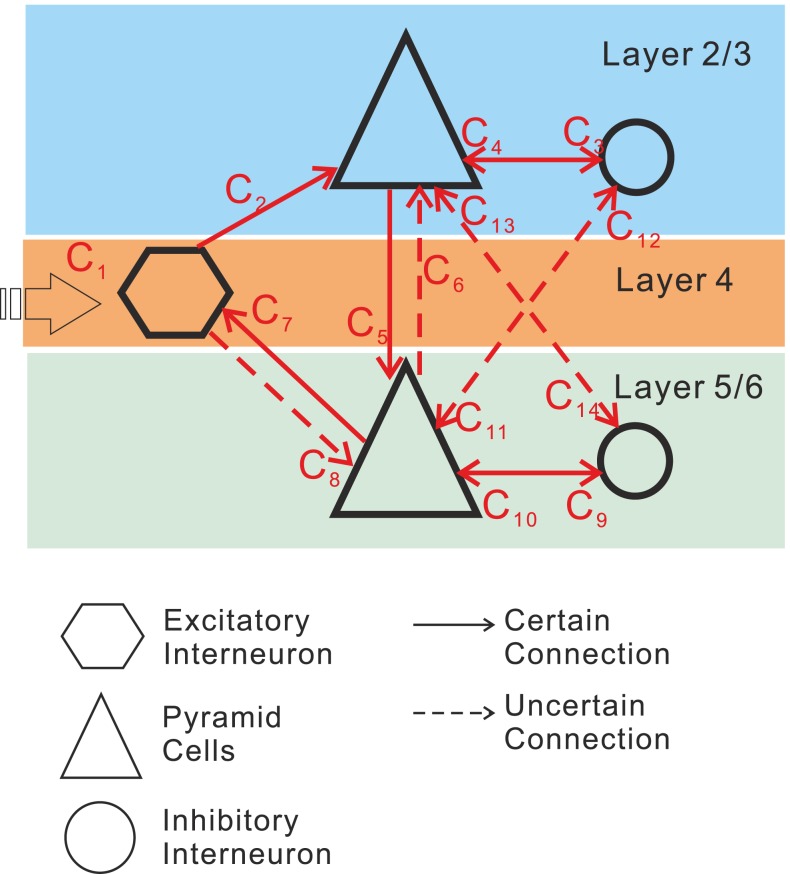
Neural mass model for a cortical source with excitatory interneurons (EIN) in layer 4, superficial pyramidal cells (sPC) in layers 2/3, deep pyramidal cells (dPC) in layers 5/6, as well as two inhibitory interneuron populations (sIIN, dIIN).

In the modeling process, our goal was not only to implement a mechanism for short-term habituation on the basis of the current knowledge from cellular research, but also to refine the very parsimonious NMM of a local cortical circuit proposed by Jansen and Rit [18]. Detailed cortical circuit models have shown that the segregated role of the different lamina of the cortex as well as the connectivity between the lamina is an important key for understanding the implementation of brain function [10]–[11], [13], [66]–[67]. Consequently, we aimed to introduce more realistic inter-laminar dynamics into the NMM. Commonly, most of the neocortex is considered to be divided into 6 distinct layers [68]. Excitatory interneurons (e.g., spiny stellate cells) are located in layer 4, pyramidal cells are found in layers 2 to 6 and inhibitory interneurons are present in all layers. We propose an extension to the Jansen and Rit model comprising 5 neural masses : one for EINs in layer 4, one for superficial pyramidal cells (sPCs) in supragranular layers 2/3, one for deep pyramidal cells (dPCs) in infragranular layers 5/6, as well as two for the supragranular and infragranular interneuron populations (sIINs & dIINs) (Fig. 2). The IIN in layer 4 are lumped into sIIN population. The laminar-specific connections among the populations were motivated by previous modeling attempts [18], [31], [69]–[70] and animal studies [71]–[72]. Our habituation model, embodying the depression and recovery processes, can be applied to all the excitatory connections to account for the stimulus specified repetition suppression. In comparison to simpler approaches [18], [27]–[28], [33], the resulting local cortical circuit model (LCCM) is more detailed and realistic with respect to laminar organization of information processing and better explains measured EEG/MEG data.

In contrast to the earlier work on auditory habituation [39] using an extended network (including A1 and the STG in both hemispheres) to simulate the entire auditory response (0–400 ms after stimulus), we focused on the N100m (70–130 ms after stimulus), which was accounted for by a single generator using MEG source reconstruction, located in the right superior middle temporal lobe near Heschl’s gyrus (for the motivation of this choice, please refer to the Discussion section). This restriction to the N100m component is motivated by the intended simplicity of the model. Taking into account the entire time course of the auditory evoked field would necessitate the inclusion of multiple neural circuits and result in a greatly enlarged parameter space, which would pose substantial challenges to the source localization and Bayesian inference procedures. Hence, in this proof-of-principle study only the dynamics of the N100m source, which captures the main features of the habituation, was modeled using our LCCM. The specific aim of this modeling was to study the effect of stimulus repetition on the intra-columnar connections between the different sub-populations and ask the following questions: How is the information processing organized with respect to the different cortical layers, and how are these connections affected by the stimuli repetition? We proposed two different hypotheses concerning the information pathways following the arrival of the bottom-up input at EINs in layer 4: (1) information follows a serial pathway, where it first ascends from layer 4 to the sPC in layers 2/3 and then goes down to the dPC in layers 5/6; (2) information follows parallel pathways, where it flows simultaneously from layer 4 to both layer 2/3 and layer 5/6, and then integrates at the dPC. We also studied the excitatory and the inhibitory cross-layer connection probability between the superficial layers and the deep layers.

In order to link our LCCM and, in particular, the generative model of habituation to observed data in an MEG experiment and explore the information pathway among the cortical layers, we used a Bayesian inference technique similar to the well-known *dynamical causal modeling* approach (DCM) [36], [73], which estimates the model parameters from the measured EEG/MEG data as well as from prior information about these parameters. The model evidence was approximated to account for model accuracy and model complexity [74]. It was used as an index for finding the most “optimized” connectional organization in the light of the data [75]. We propose a new technique for the formulation of priors for the connectivity parameters, which allows for accommodating larger portions of the model space within a single model that can be specified by fitting to the data.

## Materials and Methods

In this section, we will first describe the generative model used in our approach, namely the LCCM based on neural masses, including the habituation mechanism of its synaptic connections. This model was used to forwardly simulate the MEG signals. Second, we will explain the model inversion procedure based on Bayesian inference, including the model selection scheme used to test our different hypotheses. Finally, we will detail the experimental design, recording procedure, basic processing, and source localization of our MEG experiment.

### Ethics Statement

The study follows the guidelines of the declaration of Helsinki and has an ethical approval of the ethics commission of the University of Leipzig.

### Generative Models

#### Neural mass model of a cortical area/source

The model was developed based on the previous work of Jansen and Rit [18]–[19] and under the assumption that a population of neurons, which are lumped together and share similar physiological properties, can be described as a neural mass (NM). In the model, two state variables were used to describe the activity of an NM: average firing rate and average membrane potential. As can be seen in the equation below, each NM receives an average firing rate, *Q(t)*, as an input and converts it into an average membrane potential, *u(t)*. This conversion is called the rate-to-potential operator, embodying the convolution of the incoming spike rate with an impulse response, *h(t)*:
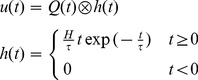
(1)


Here, *H* denotes the average synaptic gain and tunes the maximum amplitude of the average membrane potential. The time constant, *τ,* is a lumped representation of the conduction time delays, synaptic time constants and dentritic time constants of all cells belonging to the mass. The kernel, *h,* can be interpreted as Green’s function of second-order ordinary differential equation, which can be further expressed as two first-order linear inhomogeneous differential equations:
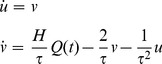
(2)


The dot over the variable indicates the time derivative ∂/∂t. The converted average membrane potential is transferred into an average firing rate and sent further to the other connected NMs. This potential-to-rate transformation is described by a sigmoid function:
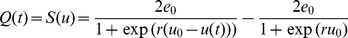
(3)


Here, *e_0_* tunes the maximum firing rate of the NM, *u_0_* is the average membrane potential for which half of the maximum firing rate is reached, and *r* is the slope of the sigmoid function. Note that we modified the original sigmoid function used by Jansen and Rit [18]–[19] to let it cross the point (*u(t) = *0, *S(u)* = 0). The motivation for this modification was to achieve a stable fixed-point, where all the states are equal to zero. The fixed-point corresponds to the system’s equilibrium or steady state. This means that all the state variables can be interpreted as the deviation from the steady state: i.e., positive and negative firing rate can be interpreted as higher or lower neural activity compared to steady state activity [36].

To simulate the neural dynamics of a single cortical area, we used five such interconnected NMs. They represent neural populations distributed in different cortical layers: superior pyramidal cells (sPC) in supragranular layers 2/3, deep pyramidal cells (dPC) in infragranular layers 5/6, excitatory interneurons (EIN) in layer 4, and inhibitory interneurons (sIIN & dIIN) in supra- and infragranular layers (Fig. 2). Based on simplified Felleman and van Essen rules for connectivity [32] and the hierarchical extension of the Jansen and Rit model by David and colleagues [31], we restricted the external input to the EIN. Note that, according to the auditory cortex studies in macaque monkeys [76], squirrel monkeys [77], and cats [78]–[79], the information flow from the ventral division of the medial geniculate complex (MGv) to the core region of the primary auditory cortex is concentrated in layers 3b and 4. Here, we did not specify the external input as thalamo-cortical input. We rather simply assumed that the external input would be a bottom-up projection from hierarchically lower areas – it could be from the thalamus or from other cortical areas. This external input was simulated by an impulse function similar to the one used in previous studies [18]–[19]:
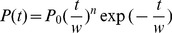
(4)


In this formula, *w* and n tune the shape and the latency of the response, while *P_0_* serves as a scaling parameter. The function represents the impulse response of the sensory pathway as proposed by Watson and Nachmias [80]. The most important parameter is the time constant *w*, which is therefore treated as a free parameter in our estimation scheme. For the exponent *n*, Jansen and colleagues [19] proposed the value 7, which we adopted as well. Note that this parameter is almost redundant with the time constant and the input gain, and is therefore not fitted in the estimation scheme. The scaling factor P_0_ was chosen 0.0064 in order to ensure that the maximum input signal is equal to the maximum firing rate of the neural masses. The intrinsic connectivity scheme of the local circuit is sketched in Fig. 2. Here, *C_1_* tunes the maximal amplitude of the input and is considered as the effective connectivity from the hierarchically lower areas to the auditory cortex. The couplings among the different NMs are described by the remaining parameters *C_i_*, controlling the maximum strength of intrinsic synaptic connections. The entire system of differential equations describing the local circuit is given by Eq. 5–10. The connections between the neural masses are, besides being controlled by the static connectivities *C_i_*, additionally controlled by the dynamic synaptic efficacies *W_i_* (Eq. 11), which embody the habituation and recovery processes. *In vivo* results from rat auditory cortex have indicated that forward suppression is due to synaptic depression, rather than inhibitory postsynaptic potentials [81]. Accordingly, we assumed that habituation would affect the excitatory synapses of our model. The specific dynamics of the synaptic efficacies *W_i_* will be explained later.

The sum of averaged membrane potentials of the sPC and dPC were assumed to be proportional to the reconstructed cortical current densities obtained by source reconstruction algorithms based on the measured EEG/MEG signals. The neuronal currents underlying EEG/MEG generation have been suggested to be produced mainly by the membrane potentials of the pyramidal cells [30], because of their asymmetric shape and parallel alignment perpendicular to the cortical surface [82].

Our NMM of a single cortical source can be described, based on Eq.2, by the following system of 26 nonlinear first-order differential equations (without habituation/recovery dynamics):

Connections to EIN, from dPC (*C_7_*) and external input:
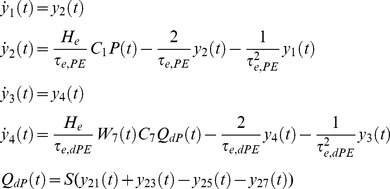
(5)


Connection to sIIN, from sPC (*C_3_*) and dPC (*C_12_*):
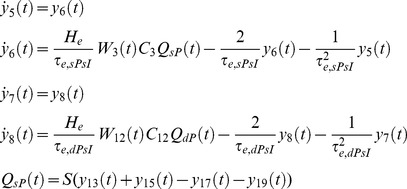
(6)


Connection to dIIN, from dPC (*C_9_*) and sPC (*C_14_*):
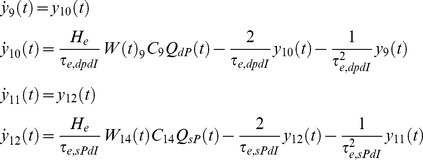
(7)


Connections to sPC, from EIN (*C_2_*), dPC (*C_6_*), sIIN (*C_4_*), dIIN (*C_13_*):
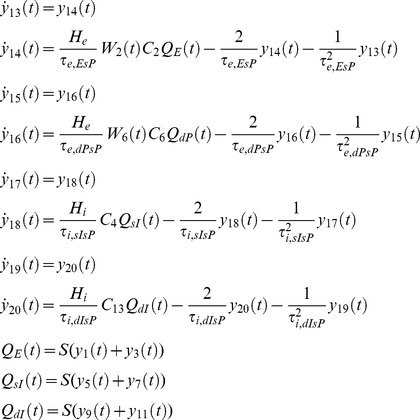
(8)


Connections to dPC, from EIN (*C_8_*), sPC (*C_5_*), sIIN (*C_11_*), dIIN (*C_10_*):
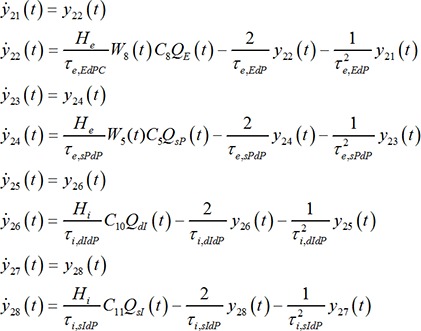
(9)


Depolarization of pyramid cells:
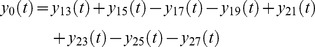
(10)


The lower case indices *e* and *i* indicate the connection types *excitatory* or *inhibitory*; *E*, *sP*, *dP*, *sI* and *dI* indicates the neural populations EIN, sPC, dPC, sIIN and dIIN, respectively. For the excitatory and inhibitory connections we used uniform synaptic gain parameter *H_e_* and *H_i_,* but connection specific time constants *τ_e,xy_* and *τ_i,xy_*. The pair of indices *xy* indicate the connection from neural population *x* to neural population *y* (*E, sP, dP, sI or dI*). This means that each connection will be uniquely characterized by three parameters: the static coupling strength *C*, the dynamic coupling strength *W* (controlled by habituation) and the time constant *τ*. The excitatory connections between EIN and PCs (*C_2,7,8_*) were motivated by previous modeling studies [18], [69]–[70], [83] as well as animal studies [71], [84]. In particular, *C_2_* (EIN→sPC) was considered as the most prominent connection in a cortical column of sensory cortex (for reviews, see [85] and reference cited therein). However, the interlaminar connectivity between the EIN in layer 4 and the sPC in layers 2/3 has been suggested to be uni-directional in the sensory cortex [71]–[72]. In terms of information flow, the anatomical existence of the connection *C_5_* (sPC→dPC) [71], [86]–[87] triggered us to propose a serial signal pathway from layer 4 up to layers 2/3 and then down to layers 5/6 [69]–[70], [83]. The reciprocal connection *C_6_* (dPC→sPC) has also been confirmed in animal studies [71], [88]–[91], but has been found to be much weaker than *C_5_* [71]. The feedback connection *C_7_* (dPC→EIN) has been reported as a projection from pyramidal cells in layer 6 to the input layer 4 in visual cortex [87] and an interaction between layer 4 spiny stellate cells and layer 5 pyramidal cells in the somatosensory cortex [92]. This connection was not mentioned in at least one animal study [71]. The direct connection from layer 4 to infragranular layers *C_8_* (EIN→dPC) was motivated by reports of a synaptic connection between layer 4 spiny stellate neurons and layer 5A pyramidal cells in rat barrel cortex [92]–[93]. As a consequence, in addition to the serial pathway hypothesis, we proposed a parallel pathway from layer 4 to layers 2/3 and to layers 5/6 [89], [94]. The connections between inhibitory interneurons and pyramidal cells *C_3_*, *C_4_*, *C_9_ ∼C_14_* were motivated by previous studies by Thomson and colleagues on rat and cat cortex [71], [95]. In summary, the LCCM structure could be simply considered as one input layer (EIN) with two output layers (sPC & dPC). The sPC targets the superficial (feedback connection) and input (feedforward connections) layers of other cortical areas. Their axons usually run through the gray matter. The dPC sends axons through the white matter and tends to target more distant cortical and subcortical areas.

Based on our *a priori* knowledge, we classified these 13 intrinsic synaptic connections into two groups. The first group of “certain” connections included EIN→sPC (*C_2_*), sPC→dPC (*C_5_*), and dPC→EIN (*C_7_*)–they form the basic laminar circuit of a column with forward (*C_2_* & *C_5_*) and backward (*C_7_*) connections, as well as intra-laminar connections between pyramidal cells and inhibitory interneurons (*C_3_*, *C_4_*, *C_9,_* and *C_10_*). The second group of “uncertain” connections, in relation to our study perspective, comprised the key connection for the parallel signal processing EIN→dPC (*C_8_*) as well as additional cross-laminar connections (*C_6_*, *C_11_ ∼C_14_*). These uncertain connections were given a zero prior expectation.

#### Short-term habituation model for NMM

**Figure 3:**
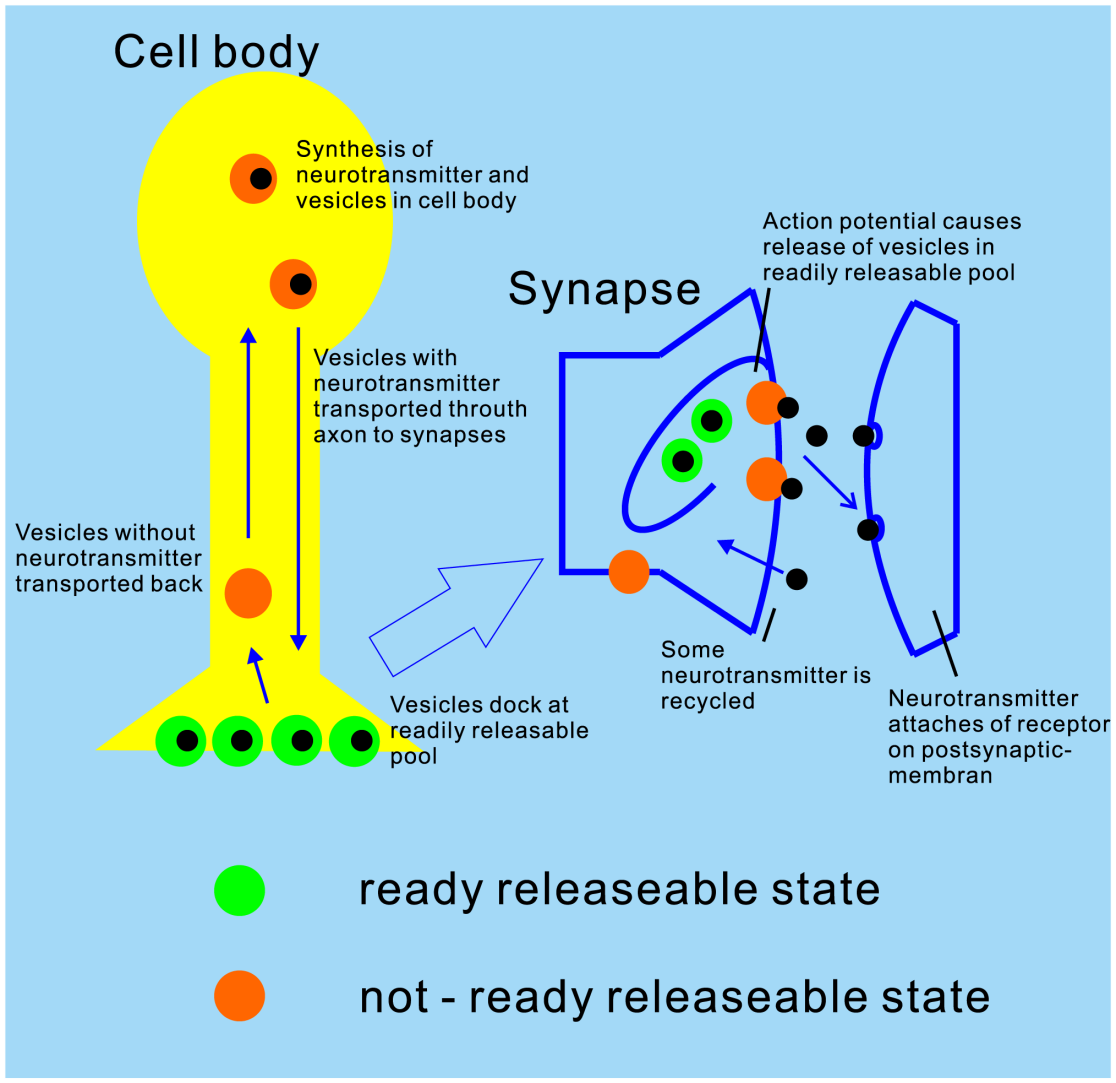
A simple sketch to show the releasing and recycling process of neural vesicles. We classify the vesicles as belonging to one of two states. Neural vesicles, which dock at the presynaptic membrane, are in readily releasable state (RR). Neural vesicles, which are under endocytosis and recycling processes are in not-readily releasable state (NRR).

Our habituation model was designed to mimic the dynamics of the synaptic depression and recovery caused by the cycle of depleting and recycling of neurotransmitters. In the framework of NMM, its relevant state variable is the dynamic synaptic efficacy *W_i_* (see Eq. 5–10). Repetitive stimulation leads to a decrease of the postsynaptic membrane potentials. This depression is due to a decrease in presynpatic transmitter release [61], [96], which can be caused by a depletion of the readily releasable pool (RRP) or by a decrease in the release probability of each docked vesicles, or both. In previous work by Sara and colleagues [63], as well as Wu and colleagues [62], three-pool models were used to simulate the vesicle kinetics underlying synaptic activity. The authors assumed that the synaptic vesicles reside in one of three pools (states): reserve pool, RRP and fused pool. The vesicles are released from the RRP after stimulation and mobilized to the fused state, and then they are endocytosised and recycled from the fused pool into the reserve pool. The vesicles in the reserve pool refill the RRP. For our purposes, we further simplified this cycle by restricting the vesicles to only two states: readily releasable (RR) and not-readily releasable (NRR), summarizing the endocytosis and recycling processes (Fig. 3). In comparison to the three-pools model, this can be regarded as lumping the fused and the reserve pools together. *A_RR_* and *A_NRR_* are the probabilities of a vesicle being in either state and the sum of them is always 1. The connectivity efficiency *W_i_* is proportional to *A_RR_*. It is equal to 1 in the steady state. According to Fig. 3, the dynamics of *A_RR_* can be described as:
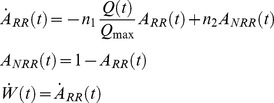
(11)


The decrease rate of the RRP, *n_1_⋅Q(t)/Q_max_*, is the depression rate depending on the current NM activity, while *n_2_* is the recycling rate of the RRP or habituation recovery rate. This description is defined for positive mean firing rates *Q(t)*. For negative *Q(t)* (see Eq. 3), the process is considered to be dominated by the recovery process alone:
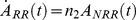
(12)


Wehr and Zador [81] reported in their *in vivo* studies that forward masking of auditory cortex cells was due to synaptic depression rather than inhibitory postsynaptic potentials (IPSPs) and Galarreta and Hestrin [97] showed that excitatory synapses depressed much more strongly than inhibitory ones. Motivated by these studies, in our model we assumed that the habituation would only affect the excitatory pathways. Moreover, because the same presynaptic neuron may have different short-term plasticity for connections to different types of target neurons [98], we used different depression and recovery rates for each excitatory connection.

### Model Inversion

Model inversion is a computational technique that uses observed data to estimate the model parameters. In our study, we used a Bayesian inversion procedure, which was similar to the one employed in the DCM framework [99]. It involved the computation and maximization of the conditional mean *η_θ|y_* of the posterior probability distribution *p(θ|y,m)* of the parameters *θ* given the model *m* and the data *y* (maximum a posteriori, MAP). According to Bayes’ rule, the posterior probability is proportional to the probability of obtaining the data given the parameters *p(y|θ)* (likelihood), multiplied by the prior probability of the parameters *p(θ)*:
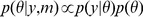
(13)


All distributions are assumed to be Gaussian. This choice is necessary to ensure that we can use the conjugate prior theorem to characterize the posterior. As all parameters and state variables are lumped over many neurons and subject to many independent random influences, the central limit theorem predicts that the distributions are at least similar to Gaussians.

An Expectation Maximization (EM) Gauss-Newton search was applied to maximize the expectation of the posterior density. This estimation procedure was described by Friston [99]. For details please see the *Text S1*.

#### Model priors

The specification of the priors was crucially important for the performance of the Bayesian inversion. The priors reflected our *a priori* information about the model structure and the parameters, based on anatomical and physiological knowledge. Because the priors were Gaussian they were specified in terms of their means and variances. The mean corresponded to our expectation on a particular parameter and the variance reflected the amount of information we had about that parameter. The parameters in our model were divided into four subsets: (i) intrinsic connectivity parameters, which reflect prior knowledge on the connections between the NMs within a cortical area; (ii) habituation parameters, which control the synaptic depression and recovery rates; (iii) synaptic and sigmoid parameters, which control the dynamics of the NMs; and (iv) input parameters, which control the time delay and dispersion of the input signal flow from subcortical or lower level cortical areas to the considered cortical area. The prior expectations and variances are listed in Tab. 1. The expectation values of the connection strengths among NMs were chosen according to proposals by by Jansen & Rit [18]. The values of synaptic gains and sigmoid parameters were chosen according to other studies [18], [33]. They were kept constant, i.e., their prior variances were zero. The dendritic time constants were assume to be 10 ms for the excitatory connections and 20 ms for the inhibitory connections [18]. We assumed that the expectation of the recovery rate would be 2 s^−1^. With this value we could ensure that, in the absence of concurrent habituation, the connection efficiency could rise from 0 to 1 within 3 seconds (5τ = 3 s, time constant τ = 600 ms). It was similar to the time constants of recovery from depression observed in the animal studies that were fitted with an exponential function: 476±104 ms (least-squares fit ± estimated fitting error *SD*) for synapses between excitatory layer 4 neurons [100], 634±96 ms [101] and 480±40 ms [102] reported for EPSPs in layer 2/3 pyramidal neurons evoked by extracellular field stimulation, 813±240 ms for connected neighboring layer 5 pyramidal neurons and 399±295 ms for layer 5 pyramidal to interneuron synapses [98]. The habituation should be far faster than the recovery, so we set this parameter to 20 s^−1^. By using this pair of habituation parameters, simulated N100m data decreased in amplitude at a similar rate as observed in other experiments.

**Table 1:** Priors of parameters.

	Expectation	Prior Type (U/I/C)
Intrinsic connection parameters		
Certain intrinsic connections		
EIN→sPC (*C_2_*)	108	U
sPC→sIIN (*C_3_*)	33.75	U
sIIN→sPC (*C_4_*)	33.75	U
sPC→dPC (*C_5_*)	135	U
dPC→EIN (*C_7_*)	135	U
dPC→dIIN (*C_9_*)	33.75	U
dIIN→dPC (*C_10_*)	33.75	U
Uncertain intrinsic connections		
dPC→sPC (*C_6_*)	0	U
EIN→dPC (*C_8_*)	0	U
sIIN→dPC (*C_11_*)	0	U
dPC→sIIN (*C_12_*)	0	U
dIIN→sPC (*C_13_*)	0	U
sPC→dIIN (*C_14_*)	0	U
Synaptic gain parameters		
*H_e_*	3.25×10^−3^[V]	C
*H_i_*	22×10^−3^[V]	C
Dendritic time constants (from NM *x* to NM *y*)		
*τ_e,xy_*	10×10^−3^[s]	U
*τ_i,xy_*	20×10^−3^[s]	U
Sigmoid parameter		
*e_0_*	2.5 [s^−1^]	C
*r*	560 [V^−1^]	C
*u_0_*	6×10^−3^[V]	C
Input parameter		
*w*	0.005[s]	I
*C_1_*	50	I
Depression and recovery rate (from NM *i* to NM *j*)		
Depression rate n*_d,ij_*	20[s^−1^]	U
Recovery rate n*_r,ij_*	2[s^−1^]	U

*Note.* Re-parameterization for uncertain connections used: *φ* = *θ^2^*, *p*(*θ*)∝N(0, 10^4^) for uninformative prior. Re-parameterization for other parameters use: *φ* = *u*⋅exp(*θ*),*u* is the expectation. The un-informative priors are *p*(*θ*)∝N(0, 1/2), the informative priors are *p*(*θ*)∝N(0, 1/16). EIN = excitatory interneurons, sPC = superficial pyramidal cells, sIIN = superficial inhibitory interneurons, dPC = deep pyramidal cells, dIIN = deep inhibitory interneurons. U = uninformative prior, I = informative prior, C = constant.

In our model, all the parameters were positive by definition. To ensure non-negativity during the parameter estimation, we re-parameterized the original model parameters in two ways. All parameters, except for the intrinsic connection parameters, had to be non-zero at all times. They were re-parameterized with *φ* = *u*⋅exp(*θ*), where *u* is the prior expectation and *θ* is the new parameter with a zero mean Gaussian prior *p*(*θ*)∝N(0,*C_θ_*). A variance of *C_θ_* = 1/2 corresponded to a rather uninformative prior and allowed the parameter to be relatively freely tuned depending on the data, while a variance of *C_θ_* = 1/16 corresponded to an informative prior and prevented the parameter from running away very far from its expectation [36]. However, this re-parameterization technique kept the model parameter away from zero. If one likes to explicitly allow a parameter to be zero, for example in the case of the intrinsic connection strengths *C_6_*, *C_8_*, *C_11_–C_14_* (i.e., the possibility that the connection does not exist at all), a different re-parameterization can be used: *φ* = *θ^2^*. Here, a variance of *C_θ_* = 10^4^ was used as uninformative prior. This type of re-parameterization yielded a true shrinkage prior behavior with respect to the original parameters, that is, the parameter (e.g., the connection strength) was assumed to be zero, unless the data provided sufficient evidence to the contrary.

### Model Comparison

We fitted the MEG data (see below) with both the J&R model (see *Table S2* for parameter configuration) and the LCCM model. Different models were compared by means of approximations of their *model evidences* [74], where the most likely model is the one with the largest evidence. Therefore, two models, *M_i_* and *M_j_*, were compared by means of the Bayes factor, *B_ij_*, computed as the ratio of the model evidences. This is often expressed in term of their logarithms:
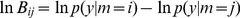
(14)


Strong evidence in favor of one model requires the difference in log-evidences to be three or more [74], [103].

### Data Acquisition and Processing

#### Experiment

Six right-handed and normal-hearing subjects (aged 18–30, 3 females) participated in the experiment. Written informed consent was obtained from all subjects prior to the experiment. The stimulation paradigm was based on previous auditory short-term habituation studies [50], [58], [64]. The subjects were binaurally stimulated via earphones with a total of 160 sequences (divided into two equal blocks of ca. 20 min duration) of ten identical tones each. The tones were 900 Hz sine waves 15 ms long (including 1.5 ms fade-in and 1.5 ms fade-out time). Within one sequence, the tones were separated by 485 ms (from offset to onset). Sequences were separated by 10 s of silence. The subjects were instructed to lie on a comfortable bed and watched a silent movie with subtitles of their own choice during the MEG recordings.

#### MEG recording

MEG was recorded with a NEUROMAG-306 system (Elekta Oy, Helsinki) with 204 planar gradiometers and 102 magnetometers. Two EOG channels (vertical, horizontal) were used to detect eye blink and eye movement artifacts. The head position relative to the sensors was monitored online with 5 Head Position Indicator (HPI) coils. The signal was digitized with a bandwidth from DC to 330 Hz and a sampling rate of 1000 Hz. The raw data were corrected using MaxFilter™ for noise contamination. MaxFilter™ is based on the Signal Space Separation (SSS) method [104], which separates the biomagnetic and external interference signals. The raw MEG data were filtered offline with a 1–20 Hz band-pass filter (4096 points, FIR), and then epoched from −100 ms to 2500 ms (first stimulus presented at 0 ms, time frame included 5 stimuli) for averaging. The time range from −100 ms to 0 ms was used for base-line correction.

#### Data preparation

**Figure 4:**
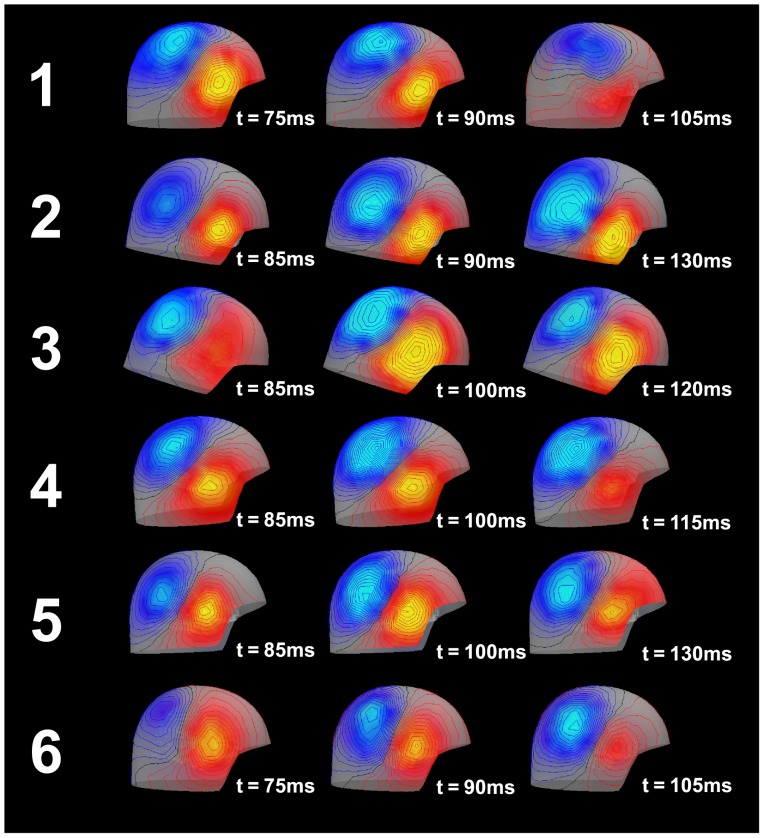
Field mapping of the observed MEG over the right hemisphere. During the development of the N100m peak, the pattern clearly suggests a single dipole located in the right superior middle temporal lobe near Heschl’s gyrus.

In order to acquire the lead field matrix for the forward modeling, a source model and a volume conductor model were prepared for each participant. For the volume conductor model, we chose a realistically shaped single-compartment *boundary element model* (BEM), which was constructed from individual anatomical MRI data. The segmentation of the MRI-data and the triangulation of the relevant surfaces were calculated using the watershed algorithm [105] in FreeSurfer (http://surfer.nmr.mgh.harvard.edu) and the MNE software [106]. We used 5120 triangles per surface. The grid spacing for the source space on the white matter surface was 5 mm. Dipole were oriented perpendicularly to the cortical surface. In the time range of the N100m (70–130 ms; see Fig. 4 for representative spatial distributions) a large proportion of the MEG can be explained by one dipolar source per hemisphere. Therefore, a representative source for the N100m generator (time windows: 70–130 ms post stimulus) in the right temporal lobe was localized using a subset of the MEG-channels on the right hemisphere (102 planar gradiometers and 51 magnetometers), by exhaustive search of a equivalent current dipole. The search area was constrained to the right Heschl’s gyrus (rHG). Note that we did not intend to find the “true” N100m generator position, but wanted to acquire a representative dipole, which captured the main features of the habituation of the N100m peak. We will discuss this issue in the *Discussion* section later. The goodness-of-fit (GoF) expressed the proportion of variance of the MEG data explained by the source, and for subjects 1 through 6 amounted to 95%, 93%, 96%, 98%, 97% and 97% at the latency of the peak of the N100m (59%, 76%, 62%, 94%, 81% and 62%, for subjects 1–6 respectively, for the entire time window). The lead field matrix of the single source for each participant was calculated using the MNE software [106]. For the Bayesian inversion, the source activity time courses were downsampled to 125 Hz and the amplitudes were normalized to their maximum.

**Figure 5:**
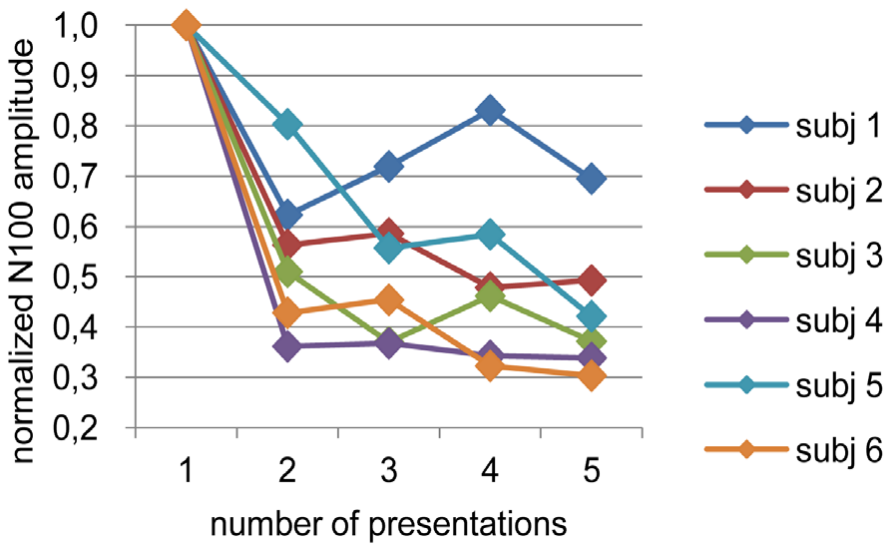
Habituation of the N100m source. The amplitudes are normalized to the responses to the first stimulus.

**Figure 6:**
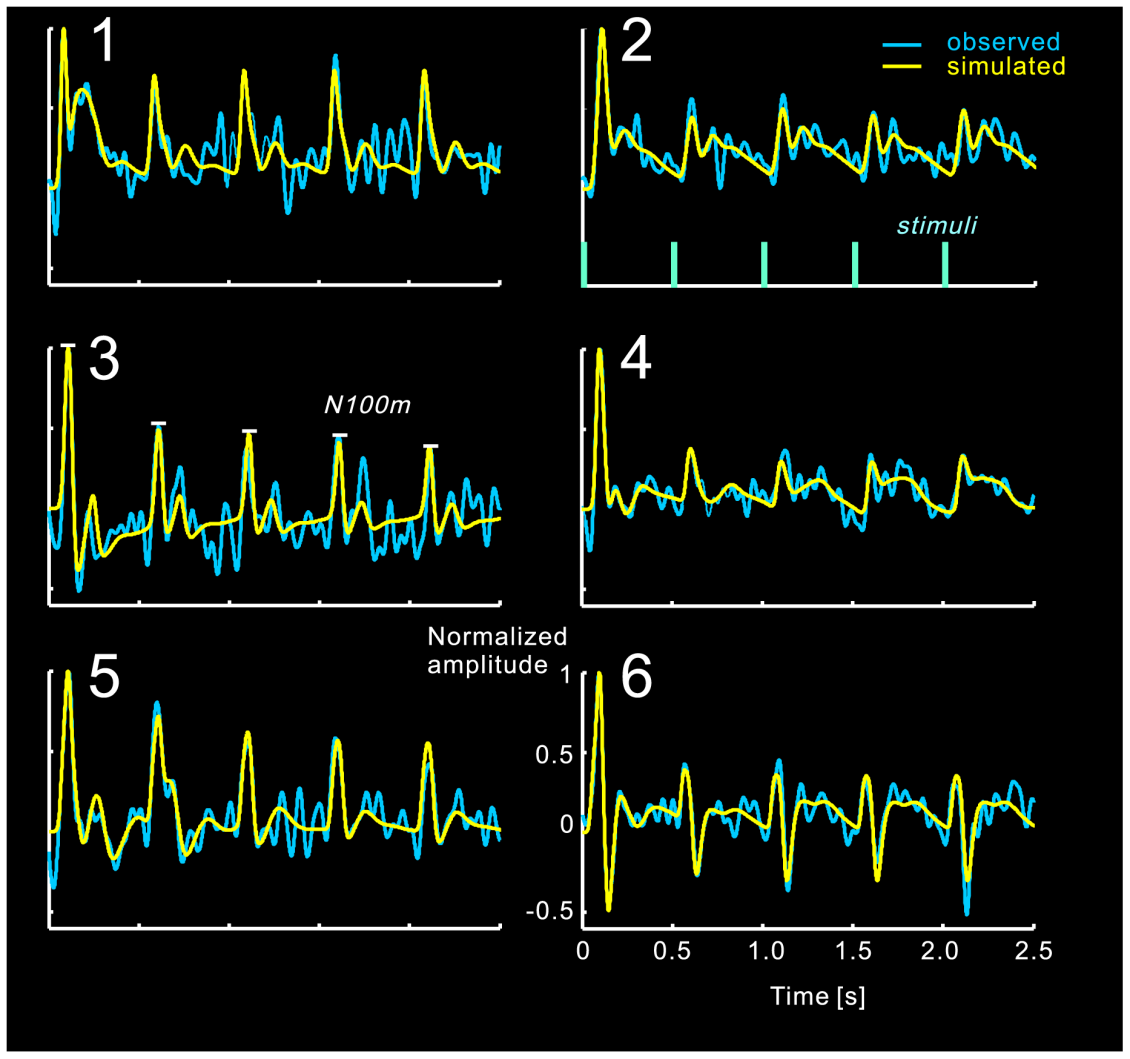
Observed and simulated (using LCCM) time courses of dipole activities.

The habituation effect was observed in all participants (N100m source) and the suppression seemed to converge after the second stimulus (Fig. 5). The field mapping of MEG over right hemisphere around N100m peak are shown in Fig. 4. The observed source activities are shown in Fig. 6.

## Results

**Figure 7:**
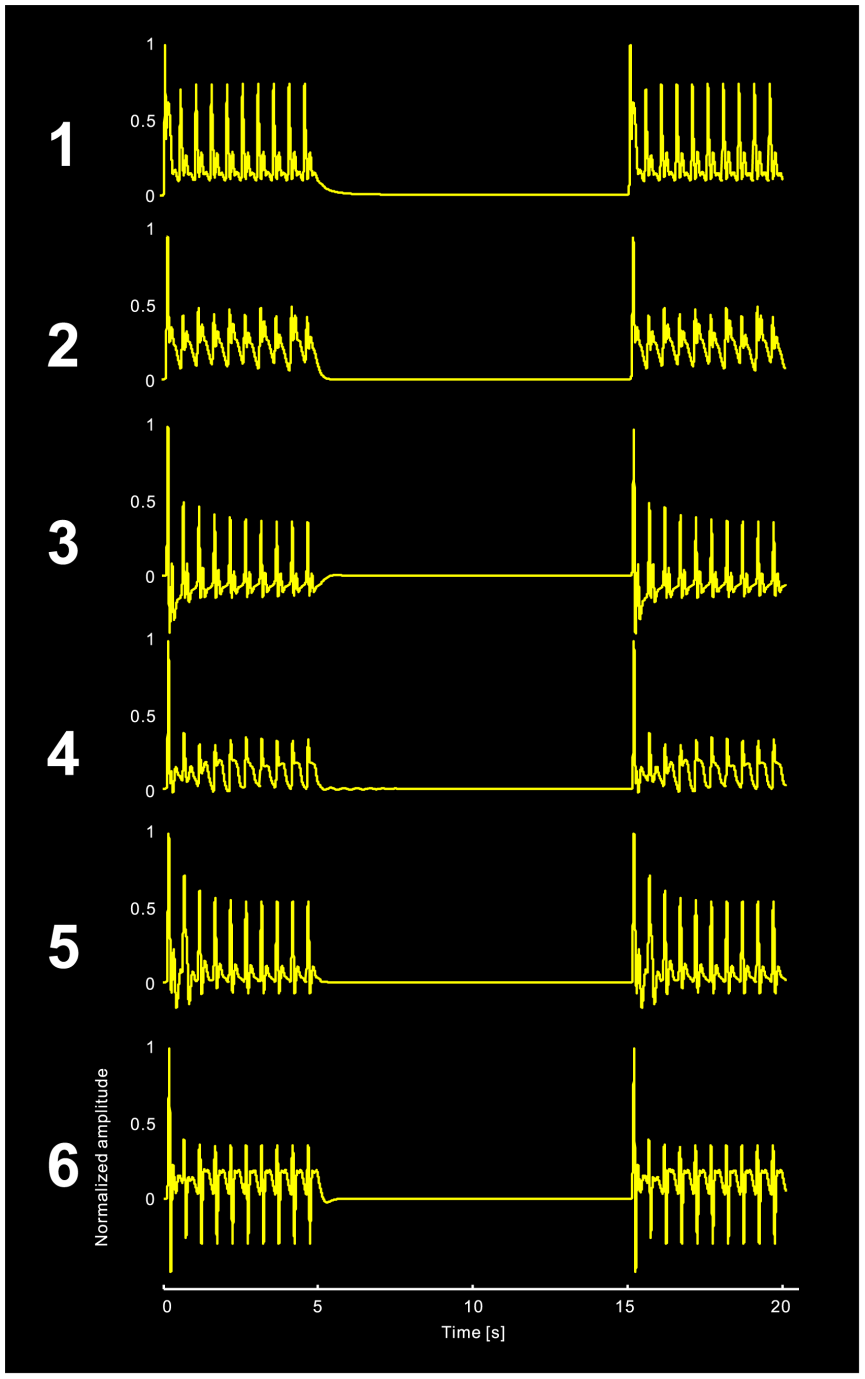
Simulated data to demonstrate that the N100m peak recovers completely during the 10 s stimulus free time.

We first compared the fitting results (goodness of fit and model evidence) of the Jansen and Rit model (JRM) and the local cortical circuit model (LCCM), at both single subject and group levels. Then we studied the effective connection probability for those connections with zero priors. Of special interest was the connection from the EIN to the dPC (*C_8_*): values near zero would support the serial signal processing hypothesis, while larger values would support the parallel signal processing hypothesis. We also studied the habituation effects for each of the estimated connections. Finally, we demonstrated that, in our model, the habituation effect depends on the inter-stimuli interval (ISI) as has been observed in previous work [64]. We show the simulated habituation processes of the source activities in Fig. 6 as well as the simulated recovery processes in Fig. 7.

### Comparison of the Local Cortical Circuit Model (LCCM) with the Jansen and Rit Model (JRM)

**Figure 8:**
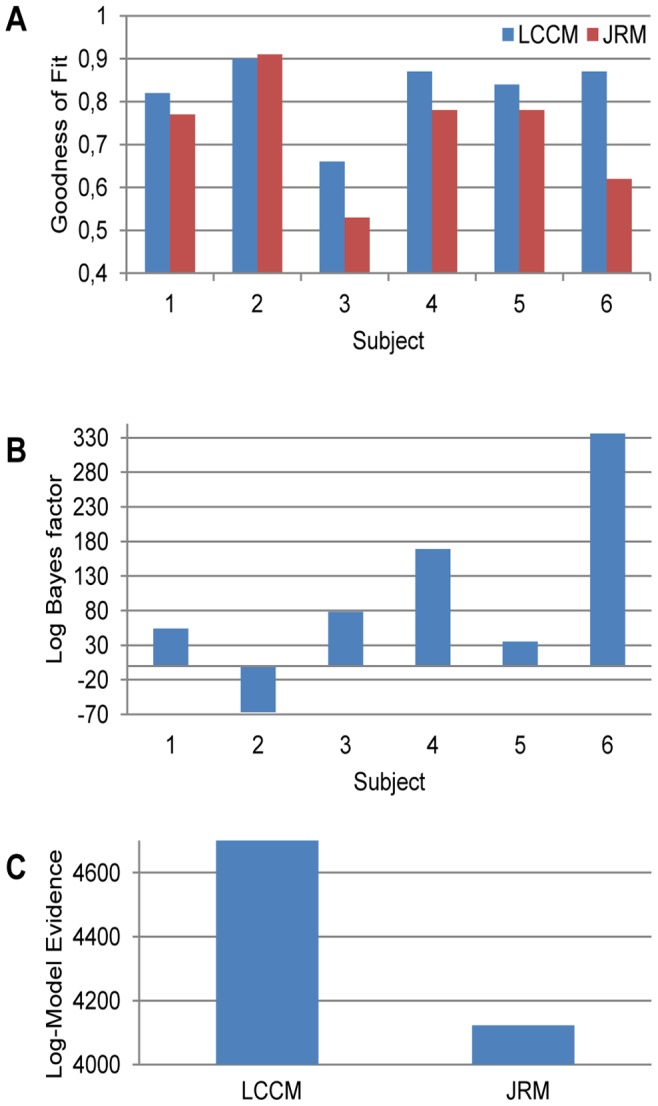
Model comparisons for LCCM and JRM. (a) Goodness of fit. (b) Log Bayes factors on individual subject level. (c) Log model evidences at group level (sum over all subjects).

We compared the goodness of the fit (Fig. 8a) and the model evidence (Fig. 8b,8c) of the LCCM and JRM. In most subjects, the LCCM yielded the better fit to the data, while for one subject (Subj. 2) there was virtually no difference. Accordingly, the model evidence, comprising both model fit and model complexity, was in favor of the JRM in that one subject, while for all other subjects there was clear evidence for the LCCM. Hence, in most cases the data supported the more detailed description of the local cortical circuit embodied in the LCCM.

### Laminar Signal Flow in a Cortical Column

We examined the six “uncertain” connections (*C_6_*, *C_8_*, *C_11_* – *C_14_*) from the LCCM of each subject. The “uncertain” connections were assumed to possibly not be necessary for the effective laminar information transfer (expressed by zero prior expectation) and this hypothesis would be rejected only if they were estimated with a non-zero value by given data. A value is considered non-zero, if the 10% tail (one sided) of the posterior distribution did not include zero. See Tab. 2 for the results. Three subjects (1, 5 & 6) supported the parallel information flow hypothesis.

**Table 2:** Estimated “uncertain“ connections. Non-zero connections are marked with “X”.

	Sub. 1	Sub. 2	Sub. 3	Sub. 4	Sub. 5	Sub. 6
EIN→dPC	x				X	X
dPC→sPC		x				
sIIN→dPC				x	X	X
dPC→sIIN						X
dIIN→sPC					X	X
sPC→dIIN	x					

*Note.* EIN = excitatory interneurons, dPC = deep pyramidal cells, sPC = superficial pyramidal cells, sIIN = superficial inhibitory interneurons, dIIN = deep inhibitory interneurons.

### Habituation of Synaptic Connections

We examined the synaptic efficacies, *W_i_*, for each existing excitatory connection at the stimulation time points (500 ms, 1000 ms, 1500 ms, and 2000 ms) (Tab. 3 and *Table S2*). The excitatory forward pathways (EIN→sPC, sPC→dPC) seem to be more strongly suppressed than the backward pathway (dPC→EIN). The parallel signal flow (EIN→dPC) seems to be little affected by the stimulus repetition (Tab. 3).

**Table 3:** Synaptic efficacies at the time point of the fifth stimulus (when the habituation is usually converged, the synaptic efficacies at the 2nd, 3th, 4th and 5th stimulus are listed in Table S2).

	Sub.1	Sub.2	Sub.3	Sub.4	Sub.5	Sub.6
EIN→dPC	0.8	–	–	–	0.97	0.94
dPC→sPC	–	0.53	–	–	–	–
dPC→sIIN	–	–	–	–	–	0,96
sPC→dIIN	0.51	–	–	–	–	–
EIN→sPC	0.32	0.41	0.49	0.32	0.47	0.33
sPC→dPC	0.56	0.86	0.57	0.48	0.8	0.72
dPC→EIN	0.56	0.96	0.8	0.96	0.9	0.95
sPC→sIIN	0.62	0.6	0.83	0.72	0.92	0.94
dPC→dIIN	0.89	0.6	0.82	0.97	0.86	0.97

*Note.* EIN = excitatory interneurons, dPC = deep pyramidal cells, sPC = superficial pyramidal cells, dIIN = deep inhibitory interneurons, sIIN = superficial inhibitory interneurons.

### Simulated ISI Effect

**Figure 9:**
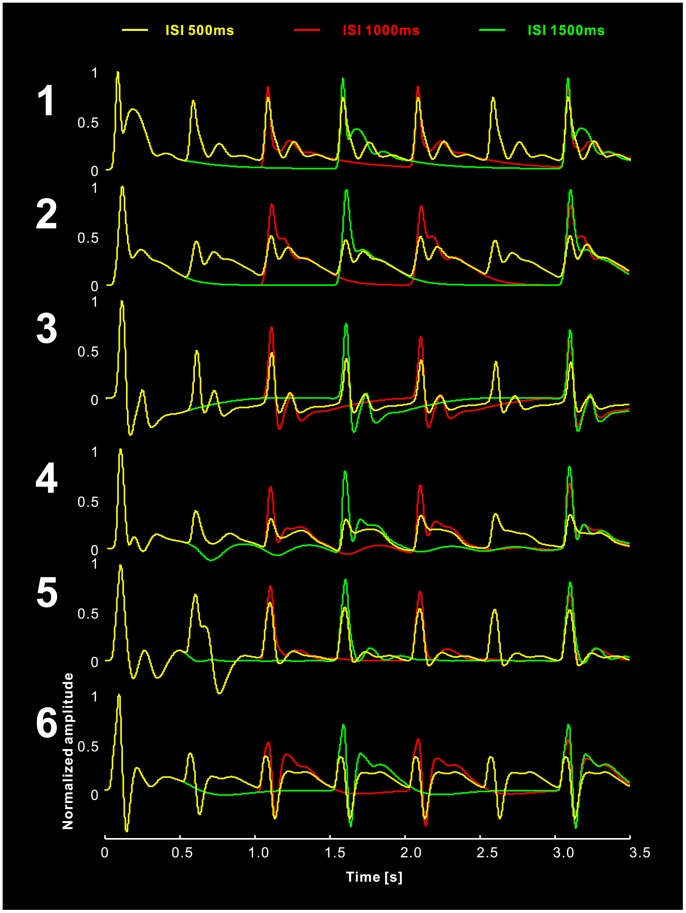
Simulated data using variable inter-stimuli interval (ISI) (500 ms, 1000 ms, and 1500 ms).

We simulated the habituation process with variable ISIs (500 ms, 1000 ms, and 1500 ms) using the LCCM. The results demonstrate stronger suppression for shorter ISIs (Fig. 9). A similar effect was reported previously [64].

## Discussion

In this piece of work, we developed a detailed neural mass model (NMM) featuring use-dependent synaptic plasticity, and used it to account for a specific component of auditory evoked fields (AEFs), namely the N100m. In particular, we aimed to explain the phenomenon of short-term habituation, which refers to the fact that a rapid succession of identical stimuli results in a reduction of the associated neural response.

The NMM approach has the advantage that it describes neural activity at the level of detail that is captured by extracranial measurements, like EEG/MEG, and at the same time maintains a certain degree of biological realism in the sense that the parameters of the model directly relate to biophysical quantities. We extended the NMM proposed by Jansen and Rit [18] by distinguishing populations in three different cortical layers (input layer 4, superficial layers 2/3 and deep layers 5/6), resulting in a local cortical circuit model (LCCM) with anatomically motivated intra- as well as inter-layer connections. The excitatory connections among the neural populations were endowed with dynamic synapses. These synapses decreased their efficacy in response to the input and recovered spontaneously. The rate of this habituation was related to the processes of exhaustion and recycling of neurotransmitters, in this case glutamate. The modeling results show that these assumptions are sufficient to reproduce the habituation effects observed in our experiment. Furthermore, the employed Bayesian inference technique allowed us to examine both model structure and model parameters. It enabled the observed data to identify the most probable signal flow circuit inside a cortical column. The results suggest that beside the main signal flow, which first ascends from input layer 4 to superficial layers and then runs down to the deep layers, there possibly exists a “short-cut” parallel input flow running directly from layer 4 into deep layers. The results also show that the excitatory signal flow from the pyramidal cells to the inhibitory interneurons seems to be preferably intra-laminar, while, in contrast, the signal flow from the inhibitory interneurons to the pyramidal cells seems to be both intra- and interlaminar. Further interesting findings were acquired through the examination of the estimated connection strengths. The feedforward connections (layer 4 -> layer 2/3 -> layer 5/6) were far more suppressed by the habituation process than the feedback connection (layer 5/6 -> layer 4). Finally, the “short-cut” parallel signal flow was only weakly affected by the habituation.

The most challenging part of the interpretation of our computational findings (in fact, the most challenging part of modeling in general) was to show that our model is reasonable in terms of the reflected level of detail and physical realism. Concerning detail, the model should be adapted to the quality and the quantity of the available data, as well as to the questions the model is supposed to answer. Physical realism concerns the interpretability of structure, state variables, and parameters of the model in terms of physically observable quantities. We believe our LCCM is a suitable candidate for modeling cortex and discuss this, with respect to the aforementioned aspects, in the following sections.

### Modeling N100m using a Single LCCM

The first question is: To what extent it is justifiable to explain the N100m response by a single dipole in the auditory cortex?

Different approaches have been used in previous studies to account for the generators of the auditory N100/N100m response in EEG/MEG observations. Zouridakis and colleagues [107] found that using a single moving dipole within the primary auditory cortex could account for the entire duration of the N100m (from about 70 ms to 150 ms after stimulus) and that during the evolution of the component, it followed a bilateral posterior-anterior, medial-lateral, superior-inferior trajectory, extending about 2 cm into the superior surface of the temporal lobes. This finding was confirmed by several other MEG studies [58], [108]. Lu and colleagues [109] postulated that it might be possible for one neural source in the primary auditory cortex to account for a short ISI response and an additional one would be needed in the auditory association cortex for the long ISI response. Näätänen and Picton [51] reviewed the previous literature on the N100 (50–150 ms after stimulus) and postulated three neural generators: one tangentially oriented to the head surface and bilaterally located in the auditory cortices, making the largest contribution to the N100 recording [110]. Due to its radial orientation the second generator (in auditory association cortex in STG) is insensitive to MEG. The third generator was found only with intracranial recordings [111]–[112]. Its location is unclear and supposed to lie somewhat posterior to the first generator. Another multi-generator approach was reported by Jääskeläinen and colleagues [56]. They found two separate sources in the anterior superior temporal gyrus (STG) and posterior STG/planum temporale (PT) contributing to the N100 by combining MEG, EEG and fMRI recordings. The posterior source activated at around 85 ms and is considered to be related to the “where” information. The anterior source activated at around 150 ms and is thought to be related to the “what” information [113].

In summary, the generation of the major component of the N100m for a series of identical (location and pitch) stimuli with short ISI might be explained by a single dipole at each time step. All dipoles are located near the primary auditory cortex and their orientations seem to be very similar [58]. Hence, they have similar leadfields and their dynamics cannot be separated easily. Consequently we decided to lump these sources together and describe them by a single LCCM.

### LCCM versus JRM

The next question: why and in what respect is the LCCM proposed in our work more biologically realistic than the classical model of Jansen and Rit?

The cortex has a clear laminar structure and neural populations in different layers are structurally and functionally different. In particular, the cortical connections are layer-dependent: forward and backward connections target different neural populations in different layers [32]: the sPC projects to the input layers and superficial layers of other cortical areas through the grey matter, while the dPC sends its axons through the white matter to more distant cortical and subcortical areas.

The JRM features only one neural mass of pyramidal cells and is, therefore, not capable of separating the different types of long-range connectivity. Using separate supra- and infragranular populations also allows a distinction to be made between different local information processing schemes (serial vs. parallel pathway) that might relate to different cognitive functions. In these respects, the LCCM constitutes an improvement in biological realism as compared to the JRM. This improvement appears relevant in light of the available MEG data, as shown by our model comparison results. We expect that the LCCM will be useful building in more extended models in the future, endowing them with the above-described advantages.

### Laminar Connections and Signal Flow

In this study, we combined MEG recordings with the LCCM in order to infer the laminar connections in auditory cortex. We postulated there to be 13 connections within and between cortical layers, divided into “certain” and “uncertain” (with logarithmic and quadratic prior, respectively) connections. The results showed that, in some subjects, besides the main serial excitatory signal flow circuit (layer 4 -> layers 2/3 -> layers 5/6), a “short-cut” parallel pathway allowed the sensory input directly from EINs to access the pyramid cells in deep layers 5/6. The functional meaning of this finding is not entirely clear. The serial and parallel pathway could be related to the “specific” and “unspecific” input; where the “unspecific” one is possibly able to bypass the superficial layer through the connection EIN→dPC [93]. Clearly, the question remains as to whether this phenomenon is universal but not visible in some subjects due to unfortunate anatomical circumstances or other peculiarities of the measurement, or if there are variable processing modes across subjects.

Another interesting finding is that half of the models need a cross-layer inhibition in order to achieve reasonable fitting results: The hierarchical signal flow descending from superficial to deep layers is, possibly, both excitatory and inhibitory (sINN→dPC) and models with a parallel pathway also send cross-layer inhibitory signals to the superficial layers (dINN→sPC).

### Modeling Habituation

In our work, we successfully reproduced the short-term habituation of the N100m and its recovery via a dynamical modification of the synaptic strength. The suppression and recovery of the synaptic connections was related to the exhaustion and refilling of the neural vesicles at the RRP. The key notion is that the brain is not a static machine and has limited resources. The brain will change its reaction to the incoming information depending on the strategies of how to assign these resources. Notice that we did not directly modify our model output with a habituation rule based on phenomenological observation, as was the case in previous work by Laxminarayan and colleagues [114] developing an NMM for rat EEG or by Petersen [100] modeling the excitatory postsynaptic potentials on single neuron level. Instead, we implemented a physiologically motivated process to generate dynamic synapses in our NMM, which increased the biological plausibility. The observed stimulus repetition related short-term habituation is only the final result of a serial dynamic process. In other words, our main purpose was not to just mimic the phenomenon of habituation (like parameterized curve fitting), but to develop a simple, yet biological plausible model, which contains sufficient detail to reproduce this aspect of real brain activity and makes testable prediction on the underlying mechanisms. These mechanisms may concern, for example, the reuptake rate of neurotransmitters, the effect of ISI, the asymmetric suppression of the synaptic connection in feedforward and feedback pathways, and the existence of a parallel bypass pathway.

### Conclusion

In this work we described a neural mass model of a local cortical circuit that features some important aspects of brain functionality that were not covered by previous neural mass models. These aspects are: (1) inclusion of activity induced synaptic plasticity, (2) inter-and intralaminar information transfer, and (3) distinction between supra- and infragranular output routes. These aspects, in particular the first one, are of crucial importance for any mechanistic explanation of brain function. Their incorporation into a mass model makes them available to modeling based on macroscopic data (like EEG or MEG), which are usually available in human experiments. We demonstrated the usefulness of the model at the example of short-term auditory habituation. Our LCCM is potentially a valuable building block for more realistic models of human cognitive function.

## Supporting Information

Table S1 **Parameter prior distributions of Jansen and Rit Model.**
(DOC)

Table S2 **Synaptic efficacies at the time point of 2nd, 3th, 4th and 5th stimulus (500 ms, 1000 ms, 1500 ms, and 2000 ms).**
(DOC)

Text S1 **Computing the Bayesian inversion procedure.**
(DOC)
