# Supplementary material for: A Realistic Neural Mass Model of the Cortex with Laminar-Specific Connections and Synaptic Plasticity – Evaluation with Auditory Habituation
Source: PLoS One. 2013 Oct 30;8(10):e77876. doi: 10.1371/journal.pone.0077876 (PMC3813749; doi:10.1371/journal.pone.0077876)
Supplement: Table S1 — Parameter prior distributions of Jansen and Rit Model. (DOC) [file pone.0077876.s001.doc]

### Supporting information S2

Table S1: Parameter prior distributions of Jansen and Rit Model, adapted from [6].

|  | Expectation | Prior Type (U/I/C) |
| --- | --- | --- |
| Intrinsic connection parameters | | |
| Certain intrinsic connections | | |
| EIN🡪PC (*C_2_*) | 108 | U |
| PC🡪EIN (*C_3_*) | 135 | U |
| PC🡪IIN (*C_4_*) | 33.75 | U |
| IIN🡪PC (*C_5_*) | 33.75 | U |
| Synaptic gain parameters |  |  |
| *H_e_* | 3.25x10^-3^[V] | C |
| *H_i_* | 22x10^-3^[V] | C |
| Dendritic time constants (from NM *x* to NM *y*) | | |
| *τ_e,xy_* | 10x10^-3^[s] | U |
| *τ_i,xy_* | 20x10^-3^[s] | U |
| Sigmoid parameter |  |  |
| *e_0_* | 2.5 [s^-1^] | C |
| *r* | 560 [V^-1^] | C |
| *u_0_* | 6x10^-3^[V] | C |
| Input parameter | | |
| *w* | 0.005[s] | I |
| *C_1_* | 100 | I |
| Depression and recovery rate (from NM *i* to NM *j*) | | |
| Depression rate n*_d,ij_* | 20[s^-1^] | U |
| Recovery rate n*_r,ij_* | 2[s^-1^] | U |

*Note.* Re-parameterization for parameters use: *φ*=*u*⋅exp(*θ*)，*u* is the expectation. The un-informative priors are *p*(*θ*)N(0, 1/2), the informative priors are *p*(*θ*)N(0, 1/16). EIN = excitatory interneurons, PC = pyramidal cells, IIN = inhibitory interneurons. U = uninformative prior, I = informative prior, C = constant.
