# Supplementary material for: A Realistic Neural Mass Model of the Cortex with Laminar-Specific Connections and Synaptic Plasticity – Evaluation with Auditory Habituation
Source: PLoS One. 2013 Oct 30;8(10):e77876. doi: 10.1371/journal.pone.0077876 (PMC3813749; doi:10.1371/journal.pone.0077876)
Supplement: Table S2 — Synaptic efficacies at the time point of 2nd, 3th, 4th and 5th stimulus (500 ms, 1000 ms, 1500 ms, and 2000 ms). (DOC) [file pone.0077876.s002.doc]

### Supporting information S3

Table S2: Synaptic efficacies at the time point of 2nd, 3th, 4th and 5th stimulus (500ms, 1000ms, 1500ms, and 2000ms).

| Subject 1 | 2nd | 3th | 4th | 5th |
| --- | --- | --- | --- | --- |
| EIN🡪dPC | 0,8 | 0,84 | 0,79 | 0,8 |
| dPC🡪sPC | --- | --- | --- | --- |
| EIN🡪sPC | 0,32 | 0,36 | 0,3 | 0,32 |
| sPC🡪dPC | 0,45 | 0,55 | 0,54 | 0,56 |
| dPC🡪EIN | 0,77 | 0,7 | 0,6 | 0,56 |
| sPC🡪dIIN | 0,41 | 0,52 | 0,49 | 0,51 |
| sPC🡪sIIN | 0,58 | 0,63 | 0,59 | 0,62 |
| dPC🡪dIIN | 0,93 | 0,92 | 0,89 | 0,89 |
| dPC🡪sIIN | --- | --- | --- | --- |

| Subject 2 | 2nd | 3th | 4th | 5th |
| --- | --- | --- | --- | --- |
| EIN🡪dPC | --- | --- | --- | --- |
| dPC🡪sPC | 0,52 | 0,53 | 0,5 | 0,53 |
| EIN🡪sPC | 0,4 | 0,41 | 0,37 | 0,41 |
| sPC🡪dPC | 0,85 | 0,86 | 0,84 | 0,86 |
| dPC🡪EIN | 0,97 | 0,96 | 0,96 | 0,96 |
| sPC🡪dIIN | --- | --- | --- | --- |
| sPC🡪sIIN | 0,61 | 0,6 | 0,57 | 0,6 |
| dPC🡪dIIN | 0,59 | 0,61 | 0,58 | 0,6 |
| dPC🡪sIIN | --- | --- | --- | --- |

| Subject 3 | 2nd | 3th | 4th | 5th |
| --- | --- | --- | --- | --- |
| EIN🡪dPC | --- | --- | --- | --- |
| dPC🡪sPC | --- | --- | --- | --- |
| EIN🡪sPC | 0,74 | 0,62 | 0,54 | 0,49 |
| sPC🡪dPC | 0,65 | 0,57 | 0,56 | 0,57 |
| dPC🡪EIN | 0,78 | 0,8 | 0,8 | 0,8 |
| sPC🡪dIIN | --- | --- | --- | --- |
| sPC🡪sIIN | 0,8 | 0,8 | 0,81 | 0,83 |
| dPC🡪dIIN | 0,78 | 0,81 | 0,81 | 0,82 |
| dPC🡪sIIN | --- | --- | --- | --- |

| Subject 4 | 2nd | 3nd | 4nd | 5nd |
| --- | --- | --- | --- | --- |
| EIN🡪dPC | --- | --- | --- | --- |
| dPC🡪sPC | --- | --- | --- | --- |
| EIN🡪sPC | 0,36 | 0,33 | 0,32 | 0,32 |
| sPC🡪dPC | 0,4 | 0,44 | 0,47 | 0,48 |
| dPC🡪EIN | 0,95 | 0,94 | 0,94 | 0,96 |
| sPC🡪dIIN | --- | --- | --- | --- |
| sPC🡪sIIN | 0,61 | 0,69 | 0,72 | 0,72 |
| dPC🡪dIIN | 0,96 | 0,95 | 0,96 | 0,97 |
| dPC🡪sIIN | --- | --- | --- | --- |

| subject 5 | 2nd | 3nd | 4nd | 5nd |
| --- | --- | --- | --- | --- |
| EIN🡪dPC | 0,96 | 0,97 | 0,97 | 0,97 |
| dPC🡪sPC | --- | --- | --- | --- |
| EIN🡪sPC | 0,65 | 0,53 | 0,49 | 0,47 |
| sPC🡪dPC | 0,83 | 0,79 | 0,79 | 0,8 |
| dPC🡪EIN | 0,87 | 0,87 | 0,89 | 0,9 |
| sPC🡪dIIN | --- | --- | --- | --- |
| sPC🡪sIIN | 0,9 | 0,91 | 0,91 | 0,92 |
| dPC🡪dIIN | 0,86 | 0,84 | 0,85 | 0,86 |
| dPC🡪sIIN | --- | --- | --- | --- |

| hb22 | 2nd | 3nd | 4nd | 5nd |
| --- | --- | --- | --- | --- |
| EIN🡪dPC | 0,9 | 0,94 | 0,94 | 0,94 |
| dPC🡪sPC | x |  |  |  |
| EIN🡪sPC | 0,3 | 0,34 | 0,33 | 0,33 |
| sPC🡪dPC | 0,58 | 0,69 | 0,7 | 0,72 |
| dPC🡪EIN | 0,94 | 0,95 | 0,95 | 0,95 |
| sPC🡪dIIN | x |  |  |  |
| sPC🡪sIIN | 0,91 | 0,95 | 0,95 | 0,94 |
| dPC🡪dIIN | 0,96 | 0,97 | 0,97 | 0,97 |
| dPC🡪sIIN | 0,95 | 0,96 | 0,96 | 0,96 |

*Note.* EIN = excitatory interneurons, dPC = deep pyramidal cells, sPC = superficial pyramidal cells, dIIN = deep inhibitory interneurons, sIIN = superficial inhibitory interneurons.
