# Supplementary material for: A Realistic Neural Mass Model of the Cortex with Laminar-Specific Connections and Synaptic Plasticity – Evaluation with Auditory Habituation
Source: PLoS One. 2013 Oct 30;8(10):e77876. doi: 10.1371/journal.pone.0077876 (PMC3813749; doi:10.1371/journal.pone.0077876)
Supplement: Text S1 — Computing the Bayesian inversion procedure. (DOC) [file pone.0077876.s003.doc]

### Supporting information S1

##### Computing posterior distribution

The observed source activity, *y*, can be computed by multiplying the inverse of the lead field matrix, *L*∈ℜ*^N×S^*, by the recorded EEG/MEG data, *y_EEG/MEG_*∈ℜ*^N×T^*. *N* is the number of channels, *S* is the number of sources and *T* is the number of time steps. We vectorized *y* by concatenating the column vectors of data from each source into a single column:

$$y=vec(L^{-1}y_{EEG/MEG})$$

The lead field matrix defines the projection from the dipolar current sources at discrete positions on the cortex to potential measurements at discrete recording site on or near the head surface. The observed source activity can be considered as the output of the generative model *h(θ)* plus a zero mean Gaussian noise *e*:

$$y=vec(h(\theta))+e$$

The associated likelihood computes as [84]:

$$\begin{matrix} p(y|\theta)=N(vec(h(\theta)),C_{e}) \\ p(y|\theta)\propto\exp\{-\frac{1}{2}(r-J(\theta-\eta_{\theta|y}))^{T}C_{e}^{-1}(r-J(\theta-\eta_{\theta|y}))\} \\ r=y-vec(h(\theta)) \\ J=\frac{\partial h(\eta_{\theta|y})}{\partial\theta} \end{matrix}$$

We assume that the noise *e* is independent over sources, i.e. the error covariances [24]:

$$C_{e}=diag(\exp(\lambda)\otimes V)$$

where *λ*∈ℜ*^S×1^* is an unknown vector of source specific variances, *V*∈ℜ*^T×T^* represents the error’s temporal autocorrelation matrix, which we assume here to be the identity matrix. Under Gaussian assumption the prior probabilities distribution *p*(*θ*)*N*(*η_θ_,C_θ_*) can be described as [84]:

$$p(\theta)\propto\exp\{-\frac{1}{2}(\theta-\eta_{\theta})^{T}C_{_{\theta}}^{-1}(\theta-\eta_{\theta})\}$$

with the expectation *η_θ_*∈ℜ*^P×1^* and the covariances *C*_θ_∈ℜ*^P×P^*, where *P* is the number of parameters. From equations (12, 16, 18), the logarithmic posterior density follows:

$$l=\ln p(\theta|y)\propto-\frac{1}{2}(r-J(\theta-\eta_{\theta|y}))^{T}C_{e}^{-1}(r-J(\theta-\eta_{\theta|y}))-\frac{1}{2}(\theta-\eta_{\theta})^{T}C_{_{\theta}}^{-1}(\theta-\eta_{\theta})$$

##### Maximum a posteriori using EM

The EM-algorithm can be summarized as [84,85]:

$$\begin{matrix} E-step \\ \frac{\partial l}{\partial\theta}(\eta_{\theta|y}^{(i)})= J^{T}C_{e}^{-1}r+C_{\theta}^{-1}(\eta_{\theta}-\eta_{_{\theta|y}}^{(i)}) \\ \frac{\partial^{2}l}{\partial\theta^{2}}(\eta_{\theta|y}^{(i)})\approx-J^{T}C_{e}^{-1}J-C_{\theta}^{-1} \\ \eta_{\theta|y}^{(i+1)}=\eta_{\theta|y}^{(i)}-(\frac{\partial^{2}l}{\partial\theta^{2}})^{-1}\frac{\partial l}{\partial\theta} \\ M-step \\ P=C_{e}^{-1}-C_{e}^{-1}J\left(J^{T}C_{e}^{-1}J\right)^{-1}J^{T}C_{_{e}}^{-1} \\ \frac{\partial F}{\partial\lambda_{j}}=-\frac{1}{2}tr\{P\frac{\partial C_{e}}{\partial\lambda_{j}}\}+\frac{1}{2}r^{T}C_{e}^{-1}\frac{\partial C_{e}}{\partial\lambda_{j}}C_{e}^{-1}r \\ E(\frac{\partial F^{2}}{\partial\lambda_{j}\partial\lambda_{k}})=-\frac{1}{2}tr\{P\frac{\partial C_{e}}{\partial\lambda_{j}}P\frac{\partial C_{e}}{\partial\lambda_{k}}\} \\ \lambda^{(i+1)}=\lambda^{(i)}-E(\frac{\partial F^{2}}{\partial\lambda_{j}\partial\lambda_{k}})\frac{\partial F}{\partial\lambda_{j}} \end{matrix}$$

##### Computing Model Evidence

This approximation combines two components: an accuracy term, which corresponds to the data fit, and a complexity term, which penalizes models with great disagreement between prior and likelihood, as well as those with large numbers of parameters [60].

$$\begin{matrix} \log p(y|m)=Accuracy(m)-Complexity(m) \\ Accuracy(m)=-\frac{1}{2}\log|C_{e}|-\frac{1}{2}r^{T}C_{e}r \\ Complexity(m)=\frac{1}{2}\log|C_{\theta}|-\frac{1}{2}\log|C_{\eta_{\theta|y}}|+\frac{1}{2}(\eta_{\theta|y}-\eta_{\theta})^{T}C_{\theta}^{-1}(\eta_{\theta|y}-\eta_{\theta}) \end{matrix}$$

Here the term *r*=*y*-*h*(*η_θ|y_*) represents the data prediction errors and the term (*η_θ|y_*-*η_θ_*) the difference between prior and posterior expectations.
